# The contribution of raised blood pressure to all-cause and cardiovascular deaths and disability-adjusted life-years (DALYs) in Australia: Analysis of global burden of disease study from 1990 to 2019

**DOI:** 10.1371/journal.pone.0297229

**Published:** 2024-02-21

**Authors:** Xiaoyue Xu, Sheikh Mohammed Shariful Islam, Markus Schlaich, Garry Jennings, Aletta E. Schutte

**Affiliations:** 1 School of Population Health, University of New South Wales, Sydney, Australia; 2 The George Institute for Global Health, University of New South Wales, Sydney, Australia; 3 Institute for Physical Activity and Nutrition, Deakin University, Melbourne, Australia; 4 Dobney Hypertension Centre, Medical School—Royal Perth Hospital Unit, Royal Perth Hospital Research Foundation, The University of Western Australia, Perth, Australia; 5 National Heart Foundation of Australia, Melbourne, Australia; University of Bergen: Universitetet i Bergen, NORWAY

## Abstract

**Aims:**

In a high-income country, Australia, it is unclear how raised systolic blood pressure (SBP) ranks among other risk factors regarding the overall and cardiovascular disease (CVD) burden, and whether the situation has changed over time.

**Methods:**

We analysed the 2019 Global Burden of Disease (GBD) data, with focus on Australia. We assessed ten leading risk factors for all-cause and CVD deaths and disability-adjusted life-years (DALYs) and compared findings with the Australian Burden of Diseases Study.

**Results:**

From 1990 to 2019, raised SBP remained the leading risk factor for attributable all-cause deaths (followed by dietary risks and tobacco use), accounting for 29,056/75,235 (95% Uncertainty Interval (UI) [24,863 to 32,915]) deaths in 1990; 21,845/76,893 [17,678 to 26,044] in 2010; and 25,498/90,393 [20,152 to 30,851] in 2019. Contributions of raised SBP to cardiovascular deaths for both sexes were 54.0% [45.8 to 61.5] in 1990, 44.0% [36.7 to 51.3] in 2010 and 43.7% [36.2 to 51.6] in 2019, respectively. The contribution of raised SBP to cardiovascular deaths declined between 1990 and 2010 but exhibited an increase in males from 2010 onwards, with figures of 52.6% [44.7 to 60.0] in 1990, 43.1% [36.0 to 50.5] in 2010 and 43.5% [35.7 to 51.4] in 2019. The contribution of raised SBP to stroke deaths and DALYs in males aged 25–49 years were higher than other age groups, in excess of 60% and increasing steeply between 2010 and 2019.

**Conclusion:**

Raised SBP continues to be the leading risk factor for all-cause and cardiovascular deaths in Australia. We urge cross-disciplinary stakeholder engagement to implement effective strategies to detect, treat and control raised blood pressure as a central priority to mitigate the CVD burden.

## Introduction

Australia’s overall health system ranks among the best in the world [1] with one of the highest life expectancies globally, namely 82.8 years [2]. Australia’s health metrics (e.g., deaths and life expectancy) have improved substantially over the past decades, but to project this trajectory into the coming years, it is important to scrutinise the trends of the leading contributors to death and disability.

Cardiovascular disease (CVD) is the leading cause of death in Australia, accounting for 1 in 4 deaths in 2019 according to the Australian Institute of Health and Welfare [3]. The recent projection highlighted the significant burden of CVD in Australia in terms of morbidity, mortality and lost revenue to healthcare system and the society over the next 10 years [4]. The latest 2019 Global Burden of Disease (GBD) findings highlight raised systolic BP (SBP) as the leading risk factor accounting for 10.8 million deaths globally, followed by tobacco use and dietary risks [5]. The global burden of raised SBP is rapidly increasing with the number of people aged 30–79 years with hypertension doubling from 1990 to 2019 [6].

BP control is crucial for addressing overall and CVD burden. According to the Non-Communicable Disease Risk Factor Collaboration in 2019, BP control rates in Australia lag behind many other high-income countries [6]: with only 38% of women and 28% of men had controlled BP in Australia, compared to 50% and 68% in Canada [6, 7]. Therefore, it is important to reprioritize BP control in Australia. In the process of reprioritization, it is crucial to understand the effects of raised SBP versus other major risk factors on the overall and CVD burden in Australia. Findings on how risk factor contributions have changed over the past decades can offer valuable insights into the effectiveness or inadequacy of past and present initiatives and priorities. These will potentially steer governmental health agendas towards effective strategies for preventing and mitigating the burden of CVD in the future.

The GBD Study provides data on multiple diseases in 195 countries and regions, including risks attributable to all-cause and cardiovascular deaths. By using summary exposure values for aggregates of risk factors, we focused on Australia and determined the ranking, and changes of ranking in leading risk factors, including raised SBP, that contributed to all-cause and cardiovascular deaths and disability-adjusted life-years (DALYs) in males and females between 1990 and 2019. We also determined the trends in the contribution of raised SBP to all-cause and cardiovascular deaths and DALYs over the past ten years in males and females by age groups. Given that the Australian Burden of Disease Study (ABDS) provides country-specific burden of disease estimates, we compared the GBD findings and ABDS 2015 data for consistency.

## Methods

We analysed descriptive epidemiological data from the GBD datasets between 1990 and 2019, managed by the Institute for Health Metrics and Evaluation [8] The GBD study is the most comprehensive global study that provides a tool to quantify health loss from 396 diseases and injuries, and 87 risk factors in 204 countries and territories based on empirical data. The GBD study estimates annual figures of country-specific disease measures, such as incidence, prevalence, mortality, years of life lost (YLLs), years lived with disability (YLDs), and DALYs [9].

Input data were extracted from censuses, household surveys, civil registration and vital statistics, disease registries, health service utilisation, disease notifications, and other sources [5, 10]. The Global Health Data Exchange (GHDx) [11], and the GBD Result Tool [12] were used to extract sex-pooled, age groups specific (0–9 years, 10–24 years, 25–49 years, 50–75 years and 75 years plus) and age-standardised all-cause and cardiovascular deaths and DALYs between 1990 and 2019. The GBD 2019 data sources are available on GHDx, with a total of 2,152 data sources available for Australia.

The detailed GBD estimation process has widely described elsewhere [5, 13, 14]. In brief, for the majority of diseases in GBD study, processed data are modelled to generate estimates of each quantity of interest by age, sex, location and year [13]. The modelling primarily using three standardised tools, namely Cause of Death Ensemble model (CODEm), spatiotemporal Gaussian process regression (ST-GPR), and DisMod-MR. CODEm was employed to estimate cause-specific death. A Bayesian meta-regression method tool (DisMod-MR) was used to generate most of the prevalence estimates. The DisMod-MR tool evaluated and pooled all available data, adjusted data for systematic biases, and generated estimates by world regions with uncertainty intervals (UIs) using Bayesian statistical methods [5, 13].

Ethics approval was not required for this study because it was based on a publicly available GBD database and did not include identified personal information.

### GBD causes and risk factors

We estimated attributable deaths and DALYs for all-causes and the CVD burden, including six main subtypes of CVD: ischemic heart disease (IHD), stroke, hypertensive heart disease, atrial fibrillation and flutter (AF), and peripheral arterial disease (PAD). GBD uses a hierarchical list with four levels of risk factors. Level 1 risk factors encompass three major categories: behavioural, environmental and occupational, and metabolic. Level 2 risk factors include 20 risks or clusters of risks within three major categories (e.g., Child and maternal malnutrition in behavioural); Level 3 include 52 risk factors within level 2 (e.g., Suboptimal breastfeeding in child and maternal malnutrition); and Level 4 includes 69 specific risk factors (e.g., Non-exclusive breastfeeding in suboptimal breastfeeding). Full list of risk factors categorized by levels have been illustrated in an earlier GBD publication [5]. To understand the major risk factors or clusters of risk, we included Level 2 risk factors for analysis. In order to compare to the Australia Burden of Disease Study, we choose to use the 10 most common risk factors including raised SBP, tobacco use, dietary risks, high low-density lipoprotein (LDL) cholesterol, high Body Mass Index (BMI), high fasting plasma glucose, kidney dysfunction, alcohol use, low physical activity and low bone mineral density. The rationale for including these ten major risk factors is based on evidence demonstrating causality and availability of exposure data according to the 2019 GBD study [5] and the Australian Burden of Disease Study [15]. At the time of the analysis, we accessed the most recent findings from the Australia Burden of Disease Study 2015, which enabled us to conduct the comparison. Estimates of attributable DALYs for a risk-outcome pair are equal to DALYs for the outcome multiplied by the population attributable fraction for the risk-outcome pair. The similar logic applies for estimation of attributable deaths [5]. For the estimation of each risk factor, the counterfactual distribution of exposure is the TMREL (Theoretical minimum risk exposure level) for that specific risk factor, while keeping other risk factors unchanged [5]. Thus, the sum of these risk-specific estimates of attributable burden can exceed 100%, including CVD [5]. The detailed methodologies of the GBD study and estimations of attributable deaths and DALYs were described previously [5, 10].

### Statistical analysis

The GBD protocol is available online [16]. Statistical, analytical, processing, and the estimation code used to generate the GBD results are also publicly available at GHDx [17]. Uncertainty intervals were estimated at several stages throughout the GBD modelling process [13]. Uncertainty for each outcome was estimated by recalculating every outcome of interest 1000 times, drawing from distributions of the sampling error around input data, corrections for measurement error, and estimates of residual non-sampling error and, in the case of cause of death estimates, model selection. Uncertainty intervals (UIs) were determined by the 2.5th and 97.5th values of the posterior distribution. Changes over time were considered statistically significant when the 95% UI of the percentage change were either positive or negative [18].

Arrow diagrams, with percentage of death and percentage change in age-standardised deaths and 95% UIs were used to present the changes of risk factors that contributed to all-cause deaths and CVD deaths between 1990 and 2019. For each risk factor, the percentage of deaths attributed to all-cause and cardiovascular deaths were ranked in descending order for the years 1990, 2010 and 2019, respectively. In cases where integers and values with one decimal point were identical, the ranking was determined using two decimal points. Two-way scatter plots using STATA 16.1 (Stata Corporation, College Station, Texas, USA) were used to present the contribution of raised SBP to age-standardised deaths and DALYs for each outcome between 2010 and 2019. Line graphs were also used to present the contribution of raised SBP to deaths and DALYs for each outcome between 2010 and 2019 by sex and age groups. We also performed basic comparisons between the GBD data and ABDS 2015 data (available on Australian Institute of Health and Welfare [19]) for consistency.

## Results

The number of all-cause deaths and DALYs has slightly increased between 1990 and 2019 for males and females (**S1A Fig**). The number of deaths from CVD (51,230, 95% Uncertainty Interval (UI) [47,545 to 53,026] in 1990, 45,146 [39,169 to 48,331] in 2014) and IHD (33,093 [30,906 to 34,278 in 1990; 23,393 [20,238 to 25,237] in 2014) decreased between 1990 and 2014, while increasing from 2015 onwards (**S1B & S1C Fig**). The changes of stroke, hypertensive heart disease, AF and PAD deaths and DALYs between 1990 and 2019 are shown in **S1E–S1G Fig**.

### Number of deaths and DALYs attributable to risk factors

**Fig 1:**
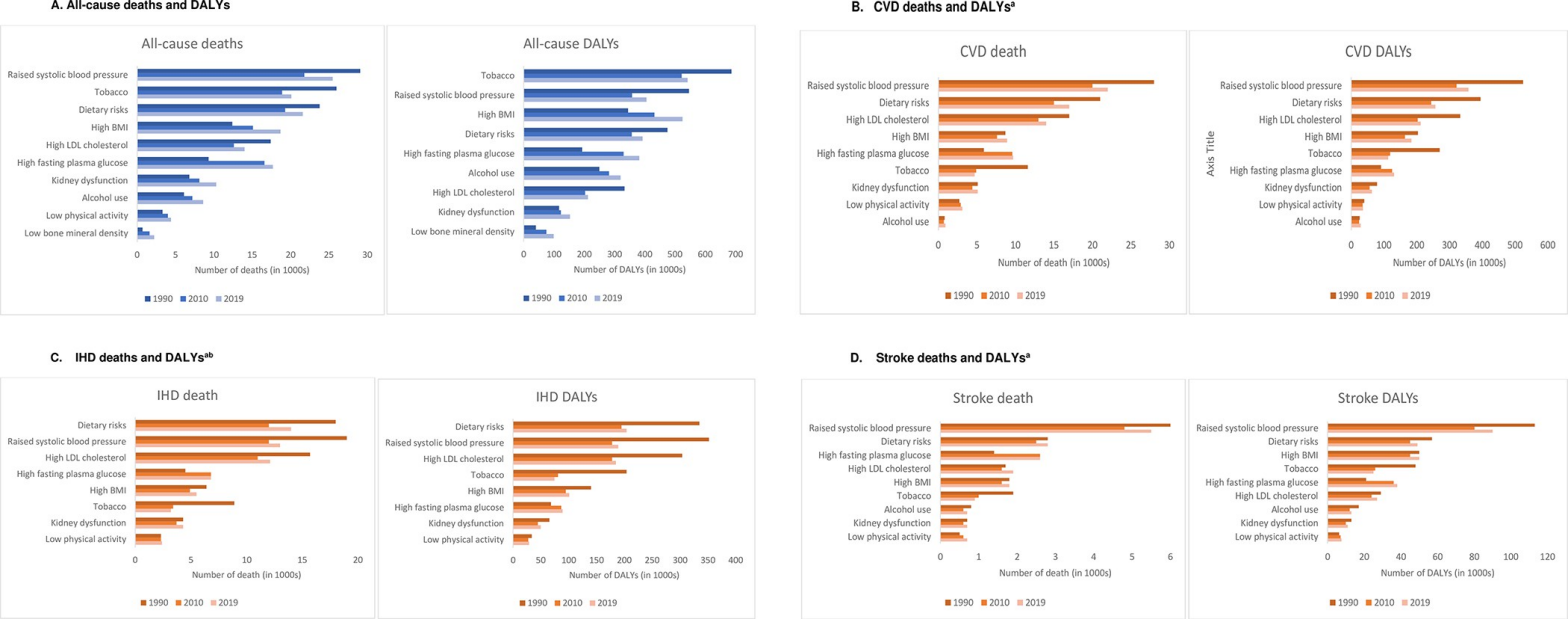
Number of deaths and DALYs attributable to risk factors in Australia between 1990 and 2019 (all ages)*. * Dietary risks include a diet low in fruit, vegetables, legumes, whole grains, nuts and seeds, milk, fiber, calcium, sea food omega-3 fatty acids and polyunsaturated fatty acids; diet high in sodium, red meat, processed meat, sugar sweetened beverages and trans fatty acids. ^a^ No data on Low bone mineral density on CVD. ^b^ Alcohol use is not included because it contributes to IHD. A. All-cause deaths and DALYs. B. CVD deaths and DALYs^a^. C. IHD deaths and DALYs^ab^. D. Stroke deaths and DALYs^a^.

Among the ten major risk factors included in this study, raised SBP is the leading risk factor for attributable all-cause deaths in Australia across all three decades, which accounted for 29,056 (95% Uncertainty Interval (UI) [24,863 to 32,915]) deaths in 1990; 21,845 [17,678 to 26044] in 2010; and 25,498 [20,152 to 30,851] in 2019 (**Fig 1A**). Raised SBP was also the leading risk factor for attributable cardiovascular deaths (27,972 [23,850 to 31,802] in 1990; 19,545 [15,582 to 23,597] in 2010; and 22,270 [17,294 to 27,425] in 2019, **Fig 1B**) and stroke deaths (6,035 [4,868 to 7,332] in 1990; 4,823 [3,609 to 6,292] in 2010; and 5,450 [3,960 to 7,158] in 2019, **Fig 1D**). The leading risk factor for IHD deaths was dietary risks (17,825 [14,672 to 20,633] in 1990; 12,467 [10,055 to 14,715] in 2010; and 13,717 [10,924 to 16,336] in 2019), followed by raised SBP (19,256 [15,678 to 23,003] in 1990; 11,561 [8,368 to 15,050] in 2010; and 12,697 [8,946 to 16,600] in 2019) (**Fig 1C**).

When viewed in terms of DALYs, raised SBP was the leading risk factor for attributable CVD (525,224 [466,689 to 586,147] in 1990; 321,752 [275,064 to 369,768] in 2010; and 357,691 [298,262 to 417,638] in 2019, **Fig 1B**) and stroke DALYs (113,026 [94,811 to 130,868] in 1990; 80,274 [65,386 to 96,070] in 2010; and 89,673 [72,413 to 108,872] in 2019, **Fig 1D**). For attributable all-cause DALYs, raised SBP ranked second (546,029 [486,082 to 607,271] in 1990; 357,783 [307,976 to 408,701] in 2010; and 406,445 [342,906 to 471,339] in 2019) behind tobacco (687,298 [647,501 to 728,873] in 1990; 521,917 [478,605 to 566,459] in 2010; and 541,539 [497,570 to 590,348] in 2019) (**Fig 1A)**. Raised SBP also ranked second for IHD DALYs (352,126 [300,589 to 403,260] in 1990; 178,186 [141,206 to 215,302] in 2010; and 188,648 [147,192 to 230,506] in 2019) behind dietary risks (335,184 [278,413 to 383,559] in 1990; 195,272 [160,985 to 225,833] in 2010; and 204,236 [167,164 to 237,980] in 2019) (**Fig 1C**).

The details of attributable deaths and DALYs to hypertensive heart disease, AF and PAD between 1990 and 2019 for risk factors are shown in **S2A–S2C Fig**. Raised SBP was the leading risk factor for attributable deaths for all these conditions across 1990 to 2019, also for attributable DALYs, except for PAD where tobacco use was the leading risk factor and raised SBP the second.

### Risk factor ranking for all-cause deaths and DALYs in 1990, 2010 and 2019

**Fig 2:**
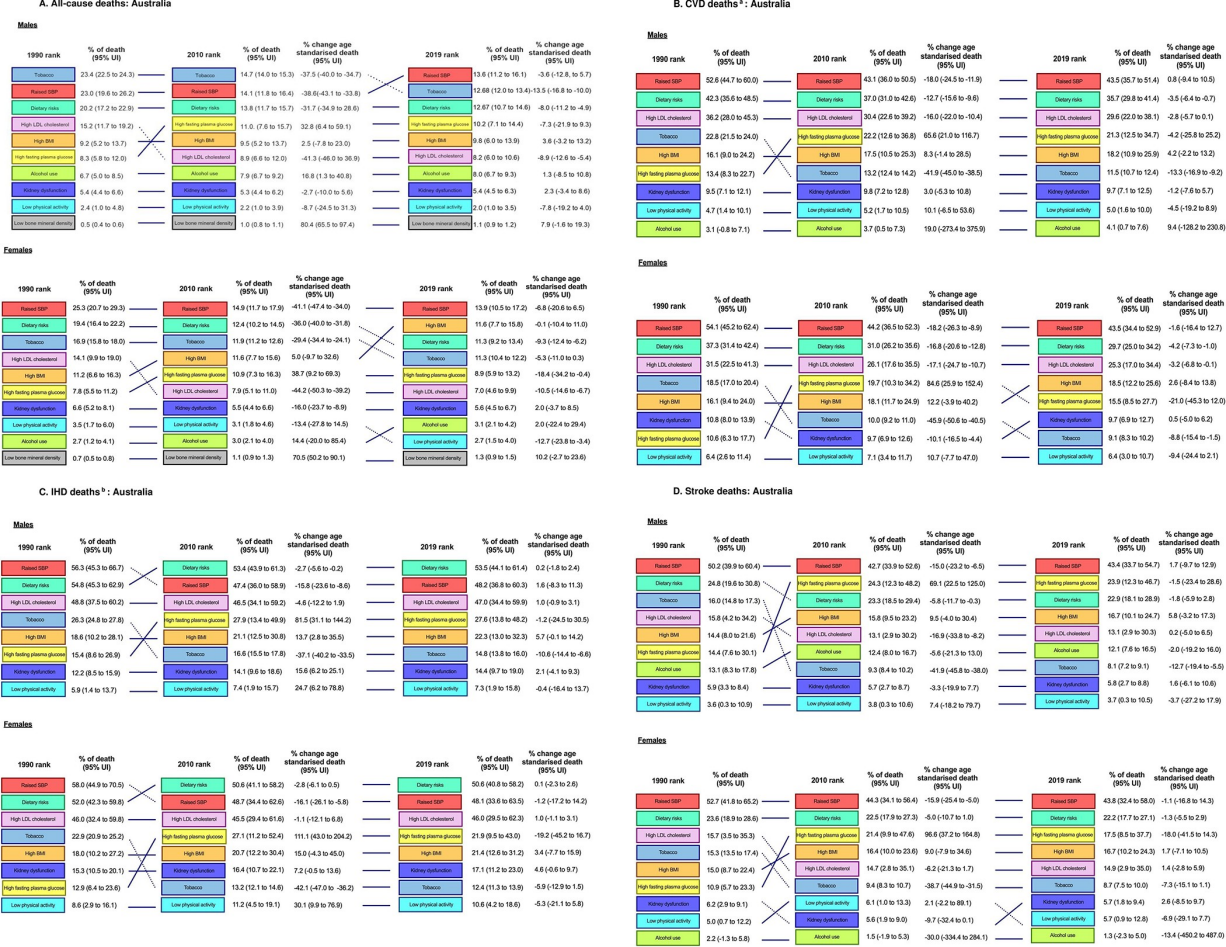
The ranking in the contribution of risk factors towards all-cause, CVD, IHD and stroke deaths by males and females in 1990, 2010, and 2019, with percentage change and 95% UI in age-standardised deaths*. ^a^ No data on Low bone mineral density on CVD. Not include alcohol in women because it negatively contributes to CVD. ^b^ Not include alcohol because it negatively contributes to IHD. **A. All-cause deaths: Australia. B. CVD deaths** ^**a**^**: Australia. C. IHD deaths** ^**b**^**: Australia. D. Stroke deaths: Australia.**

The ranking in the contribution of risk factors towards all-cause, CVD, IHD and stroke deaths in 1990, 2010, and 2019 for both sexes are shown in **S3 Fig**, and sex-specific data are shown in **Fig 2**. From 1990 to 2019 the contribution of raised SBP to all-cause deaths remained the leading risk factor for all-cause deaths in females (**Fig 2A**). Tobacco was ranked first for males in 1990 and 2010 but in 2019 raised SBP was ranked first for males. The risk of high BMI increased rapidly in ranking in females across the years, ranked from fifth in 1990 to second in 2019. High LDL cholesterol dropped from fourth in 1990 to sixth in 2019 (**Fig 2A**).

Risk factor ranking for both sexes and sex-specific DALYs are shown in **S4 Fig**. The contribution of raised SBP to all-cause DALYs decreased in males across years, which ranked second in 1990 and dropped to fifth in 2019. Raised SBP contributing to all-cause DALYs in females remained within the top three risk factors in 2019 (**S4A Fig**). In comparison, tobacco was the leading risk factor contributing to all-cause DALYs in in males across all years (**S4A Fig**). High BMI contributing to all-cause DALYs increased rapidly in males and females across the years, in particular females, which ranked from fourth in 1990 to the first in 2019.

When comparing the GBD estimates to the ABDS 2015 data, the contributions of leading risk factors to all-cause DALYs were generally consistent with our results, with tobacco and high BMI being the leading risk factors contributing to all-cause DALYs (**S1A Table**).

### Risk factor ranking for cardiovascular deaths and DALYs

The contributions of three leading risk factors, namely SBP, dietary risks and high LDL cholesterol decreased over time, but the rankings were consistent in males and females across the years (**Fig 2B**). The contribution of tobacco to cardiovascular deaths decreased over time particularly in females, which dropped from ranked fourth in 1990 to seventh in 2019 (**Fig 2B**). The leading risk factors that contributed to CVD DALYs (**S4B Fig**) were raised SBP followed by dietary risks in males and females across all years. This result was consistent with ABDS 2015 data that dietary risks and raised SBP were the two leading risk factors contributing to CVD DALYs (**S1A Table**).

### Risk factor ranking for deaths and DALYs due to IHD

The ranking of dietary risks and raised SBP remained the first and second ranked risk factors contributing to IHD deaths across 1990 to 2019 for males and females. The ranking for tobacco decreased from ranked fourth in 1990 for both males and females to sixth for males and seventh for females in 2019. (**Fig 2C**). The top three risk factors that contributed to IHD DALYs for males and females were dietary risks, raised SBP and high LDL cholesterol across the years (**S4C Fig**). This result was also consistent with ABDS 2015 data (**S1B Table**).

### Risk factor ranking for deaths and DALYs due to stroke

Across all years, raised SBP remained the leading risk factor contributing to stroke deaths for males and females (**Fig 2D)**. The contribution of fasting plasma glucose increased from ranked sixth in 1990 to the second in 2019 for males and third for females. Similar to IHD deaths, tobacco’s ranking decreased from ranked third in 1990 to seventh in 2019 in males, and from ranked fourth in 1990 to sixth in 2019 in females (**Fig 2D**). For stroke DALYs, raised SBP remained the leading risk factor for both males and females across the three decades (**S4D Fig**). This result was consistent with the ABDS 2015 data (**S1B Table**).

### Risk factor ranking for deaths and DALYs due to hypertensive heart disease, AF and PAD

Overall, raised SBP remained the top risk factor contributing to hypertensive heart disease, AF deaths and DALYs in males and females across all years (**S5A, S5B Fig**). In recent years, raised SBP and high fasting plasma glucose were ranked first and second in their contributions to PAD deaths (**S5C Fig**). These results were also consistent with the ABDS 2015 data, where raised SBP was the leading risk factor contributing to hypertensive heart disease, AF and PAD deaths and DALYs (**S1B Table**).

### Raised SBP as a risk factor from 2010 to 2019

**Fig 3:**
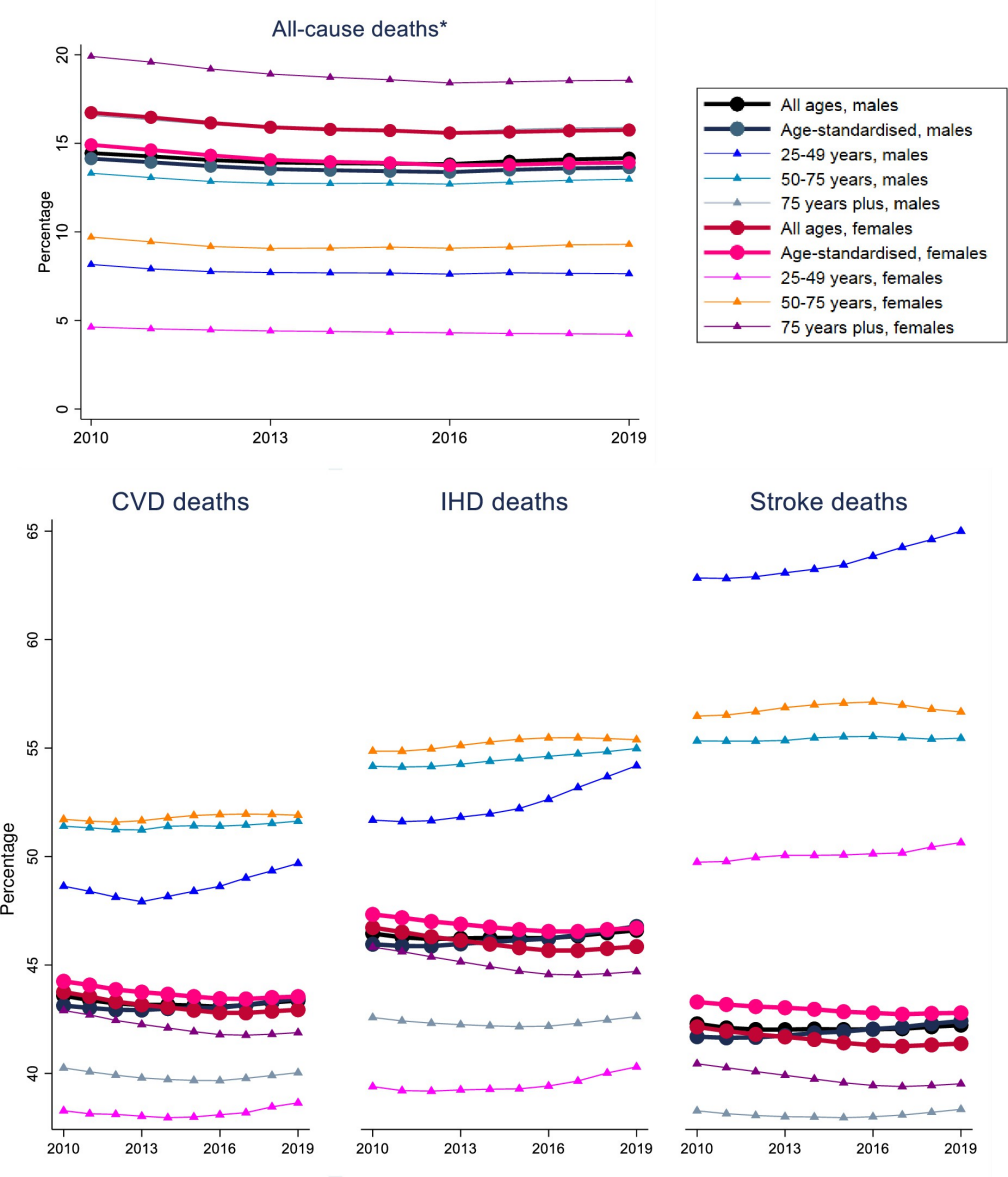
The contribution of raised systolic blood pressure to all-cause, CVD, IHD and stroke deaths between 2010 and 2019 by sex and age groups^¶^. ^**¶**^Not including age group 10–24 years for all-cause and CVD because the value tends to be 0. No data available for males and females aged 10–24 years for IHD and stroke. *The line of 75 years plus for males for all-cause deaths is overlapping with the line of all ages for females.

Given that raised SBP is the leading risk factor for all-cause and cardiovascular deaths, and the changes tended to plateau recently, we specifically examined the contribution of raised SBP to all-cause, CVD, IHD and stroke deaths and DALYs between 2010 and 2019 by sex and age groups (**Fig 3**). The contribution of raised SBP to all-cause deaths and DALYs was the highest in persons aged 75 years plus, particularly females, while being the lowest in persons aged 10–24 years. The contribution of raised SBP to CVD, IHD deaths and DALYs in males and females aged 50–74 years were higher than in other age groups (**Fig 3, S6 Fig**), with similar profiles for AF and PAD (**S7 Fig**). The contribution of raised SBP to stroke deaths and DALYs in males aged 25–49 years were higher than other age groups, in excess of 60% and increasing steeply over time. A similar but less steep upward trend were observed for females aged 25–49 years (**Fig 3, S6 Fig**).

## Discussion

The contribution of raised SBP to all-cause and CVD deaths declined substantially from 1990 to 2019 in Australia, especially during the first two decades. The Australian health system clearly improved across the past three decades as evidenced by the GBD 2017 Healthcare Access and Quality Index, where Australia ranked fifth globally [1]. According to the 2020 Bloomberg Global Health Index, Australia ranked seventh globally [20], reflecting that Australia’s health performance remains one of the best in the world. However, raised SBP persisted as the leading risk factor for all-cause and CVD deaths, with some concerning trends in raised BP contributing to particularly stroke in younger males.

Our results align with 2019 GBD global data which highlighted raised SBP as the leading risk factor for attributable deaths and DALYs globally [5]. It is widely acknowledged that raised BP or hypertension is more common in low- and middle-income countries [21], potentially resulting in raised BP being overlooked as an area for action in high-income countries [22]. According to the Australian Bureau of Statistics 2017–2018 National Health Survey, it is estimated that over one in three (34%) adults aged 18 years and over have hypertension [23]. The prevalence is higher among older individuals, with 44% of those aged 75–84 years and 47% of those aged 85 years and above experienced elevated BP [23, 24].

Several aspects of the hypertension cascade, namely awareness, treatment and control figures are low in Australia. The 2019 May Measurement Month (MMM) BP awareness campaign including 2,877 Australians who were screened for hypertension found that less than half with raised BP were aware of it [25], and 59% of those with hypertension were untreated [25]. The awareness and treatment of BP in young adults are even worse, with 97% of people aged 18–34 years who had measured raised BP being unaware of their condition and untreated, compared to 57% in those aged 75 years and over [26, 27]. The rate of uncontrolled BP in Australia is notably high at 68%, regardless of medication use, in contrast to the global rate of 38% [23, 25, 28]. Reflecting on the leading contribution of raised BP towards all-cause and cardiovascular deaths and DALYs in Australia, these numbers clearly emphasise the need to prioritise actions to improve the management of raised BP in Australia.

Apart from the focus of our study and the wide acknowledgement that raised BP is the leading risk factor for CVD and premature death worldwide [24, 29], raised BP also pose risks beyond CVD outcomes. For example, raised BP leads to cognitive decline [30], and poses an increased risk for coronavirus disease 2019 (COVID-19) and associated adverse outcomes [31]. A population-based reduction of BP would thus have wide-ranging benefits.

Although Australia’s Health 2020 Data Insights report [32] acknowledged the importance of BP as a determinant of health and lists hypertension as a potentially modifiable condition to CVD that leads to hospitalisation and cause of death, there seems to be no priority to address BP control in Australia. Recently other high-income countries have recognised that action is necessary [28, 33]. For instance, the United States proposed a *Call to Action* in 2020 by outlining a national roadmap to “make hypertension control a national priority” with a goal of improving current control rates of 44% to a rate of 70% [33].

BP is often considered to be an intermediate element in the context of public health interventions. These interventions promote lifestyle changes such as a healthy diet and increased physical activity to achieve better health and cardiovascular outcomes [34]. Despite the strong association between BP and CVD outcomes, interventions with a specific focus on improving BP control remain scarce in Australia. In addition, with the absence of a defined priority to improve BP control within the national health agenda, limited efforts and actions have been undertaken with aiming to reduce BP in the past. This notion aligns with our findings of no recent improvement in the contribution of SBP to health outcomes.

Similarly, the results from the NCD Risk Factor Collaboration confirm minimal progress in BP control rates in Australia over the past decade [6]. Collectively, these findings re-emphasise the importance to increase BP awareness, treatment and control rates and sparked us to publish a recent *Call to Action* for concerted efforts with multiple stakeholders to transform BP management strategies in Australia [28]. Key clinical and public health interventions to improve the prevention, early detection, treatment and control of raised BP would lead to significant health and economic gains [28]. An estimation of the burden of hypertension in Australia in terms of productivity lost over the working lifetime has shown that a 25% reduction in hypertension prevalence, could save 37,000 lives and return AUD$34.3 billion in gross domestic product to the Australian economy [27], whereas the adequate treatment and control hypertension would save AUD$91.6 billion [27].

Our analysis highlights that raised SBP contributed to over 50% deaths for CVD, IHD and stroke in the 50–75 age group for both males and females. This result emphasizes the significance of treating hypertension within this age group, as it could subsequently lead to a reduction in cardiovascular deaths. The release of the 2023 Australian Cardiovascular Risk Guidelines is therefore timely and ideal in targeting all people aged 45 to 79 years for CVD risk estimation and treatment [35]. Our findings also highlight males aged 25–49 years require additional attention in controlling raised BP, as this age group exhibits a notable and escalating contribution of raised SBP to CVD, IHD, and particularly stroke. According to data from the Australian Stroke Clinical Registry, one in every four strokes occurs in a person less than 65 years in Australia [36, 37]. The number of young strokes is expected to keep rising if these trends continue and are left unchecked [38]. This result is consistent with the GBD Stroke Collaborators noting the significant increase in stroke incidence in people aged 20–64 years globally in the past decades [39, 40], with raised BP being the most common contributor [9]. Our results highlight the importance of early BP screening in young adults, and in some respects challenge the limitations of absolute cardiovascular risk assessment that is relevant only to adults aged 45 years and older.

In addition, we found the ranking of other risk factors, in particular high fasting glucose levels, to demonstrate an upward trend in its contribution to all-cause and cardiovascular deaths across the three decades. It is thus important to implement strategies that would reduce overall cardiovascular risk–which may be mutually beneficial in reducing SBP as well as other risk factors such as obesity, fasting glucose and low physical activity.

This study is subject to the limitations of the GBD methodology, which have been described previously [5, 10, 41]. For example, out-of-sample predictive validity of the modelling efforts when primary data is not available [10] with uncertainty maybe underestimated [41]. Data sources for the GBD Australia included different population-based studies which may lead to compositional bias of national estimates, although most data sources included in Australia are at a national level (available on GHDx [42]) and GBD adjusts variance and weighting to reflect this possibility [41]. We also found that our main findings were aligned with the ABDS 2015 findings [43], implying consistency of the data sources. Lastly, in this paper, our emphasis was directed towards the ten major risk factors associated with both all-cause and cardiovascular mortality. However, it is worth acknowledging that there is a possibility of overlooking other potential risk factors.

In conclusion, raised SBP remained the leading risk factor for all-cause and CVD deaths in Australia across the past three decades. The contributions of raised SBP towards the CVD burden reduced substantially from 1990 to 2010 but tended to stabilise between 2010 and 2013, followed by a slight upward trajectory. A concerning rise in the contribution of raised SBP towards stroke in young males, calls for targeted action. Our findings provide valuable perspectives on the efficacy or shortcomings of past and present initiatives and priorities in Australia. These insights have the potential to guide governmental health agendas toward effective strategies for preventing and mitigating the burden of all-cause and CVD in the future. Specifically, our findings furnish evidence to support actions to improve the prevention, detection, treatment and control of raised BP, aiming to substantially reduce all-cause and cardiovascular deaths in Australia in the next decade.

## Supporting information

S1 Fig The trend for total number of all-cause and CVD deaths and DALYs by sexes from 1990 and 2019. (DOCX)

S2 Fig Number of deaths and DALYs attributable to risk factors for hypertensive heart disease, AF, and PAD in Australia between 1990 and 2019 (all ages). ^a^ No data available for the risk of high fasting plasma glucose, high LDL cholesterol, low bone mineral density, kidney dysfunction, tobacco and low physical activity for hypertensive heart disease. ^b^ No data available for the risk of high fasting plasma glucose, high LDL cholesterol, low bone mineral density, kidney dysfunction, tobacco and low physical activity for AF.^c^ No data available for the risk of high LDL cholesterol, high BMI, low bone mineral density, alcohol use, and low physical activity for PAD.
(DOCX)

S3 Fig The ranking in the contribution of risk factors towards all-cause, CVD, IHD and stroke deaths in 1990, 2010, and 2019, with percentage change and 95% UI in age-standardised deaths. ^a^ No data on Low bone mineral density on CVD. Not include alcohol in females because it negatively contributes to CVD. ^**b**^ Not include alcohol because it negatively contributes to IHD.
(DOCX)

S4 Fig The ranking in the contribution of risk factors to all-cause, CVD, IHD and stroke DALYs in 1990, 2010, and 2019, with percentage change and 95% UI in age standardised DALYs. ^a^ No data on Low bone mineral density on CVD. Not include alcohol in women because it negatively contributes to CVD.^**b**^ Not include alcohol because it negatively contributes to IHD.
(DOCX)

S5 Fig The ranking in the contribution of risk factors towards hypertensive heart disease, AF, and PAD deaths and DALYs in 1990, 2010, and 2019, with percentage change and 95% UI in age-standardised deaths and DALYs. ^a^ No data available for the risk of high fasting plasma glucose, high LDL cholesterol, low bone mineral density, kidney dysfunction, tobacco and low physical activity for hypertensive heart disease. ^b^ No data available for the risk of high fasting plasma glucose, high LDL cholesterol, low bone mineral density, kidney dysfunction, tobacco and low physical activity for AF. ^c^ No data available for the risk of high LDL cholesterol, high BMI, low bone mineral density, alcohol use, and low physical activity for PAD.
(DOCX)

S6 Fig The contribution of raised systolic blood pressure to all-cause, CVD, IHD and stroke DALYs between 2010 and 2019 by sex and age groups*. *Not including aged group 10–24 years for all-cause and CVD because the value tends to be 0. No data available for males and females aged 10–24 years for IHD and stroke.
(DOCX)

S7 Fig The contribution of high systolic blood pressure to AF and PAD deaths and DALYs between 2010 and 2019 by sex and age groups*. *No data available for males and females aged 10–24 years for AF and PAD.
(DOCX)

S1A Table Proportion of disease DALY due to top risk factors, Australia Burden of Disease Study 2015*. *Data source: https://www.aihw.gov.au/reports/burden-of-disease/interactive-data-risk-factor-burden/contents/overview.
(DOCX)

S1B Table Proportion of each CVD DALY due to risk factors by sex, Australia Burden of Disease Study 2015*. *Data source: https://www.aihw.gov.au/reports/burden-of-disease/interactive-data-risk-factor-burden/contents/diseases-and-associated-risk-factors.
(DOCX)
