# Supplementary material for: The contribution of raised blood pressure to all-cause and cardiovascular deaths and disability-adjusted life-years (DALYs) in Australia: Analysis of global burden of disease study from 1990 to 2019
Source: PLoS One. 2024 Feb 21;19(2):e0297229. doi: 10.1371/journal.pone.0297229 (PMC10881002; doi:10.1371/journal.pone.0297229)
Supplement: S1 Fig — (DOCX) [file pone.0297229.s001.docx]

**Supplementary Figure 1. The trend for total number of all-cause and CVD deaths and DALYs by sexes from 1990 and 2019**

1. **All-cause deaths and DALYs**


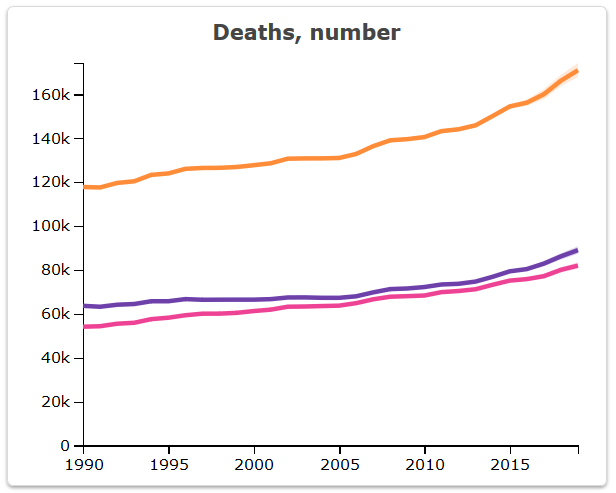

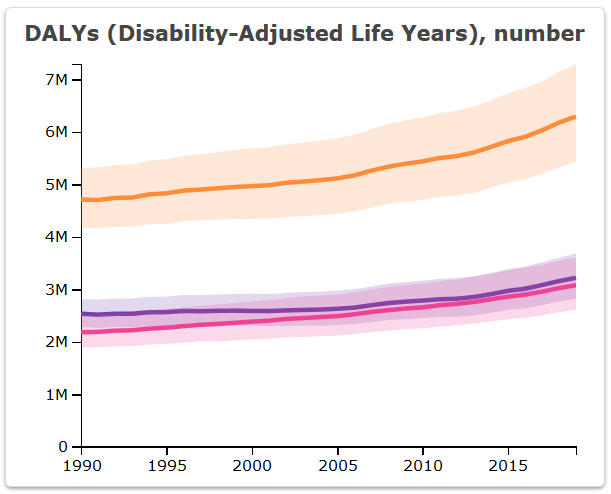


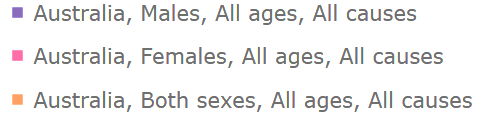


1. **Cardiovascular deaths and DALYs**


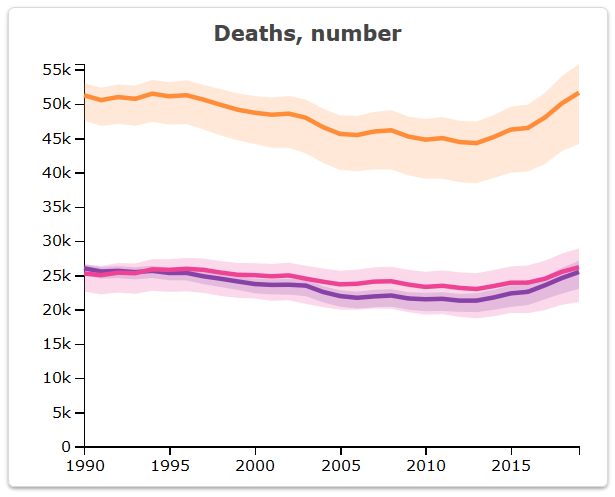

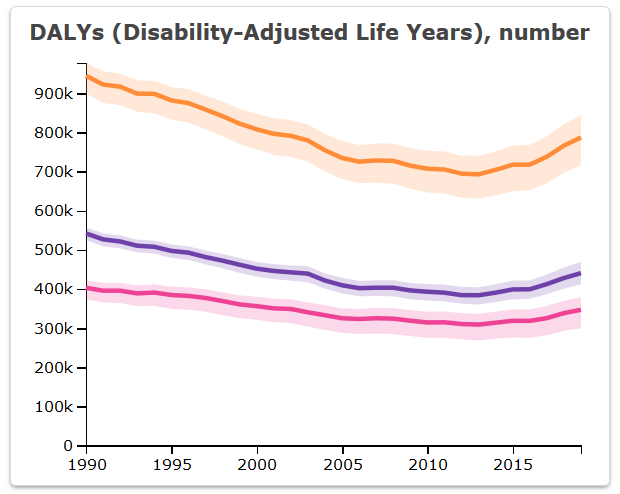


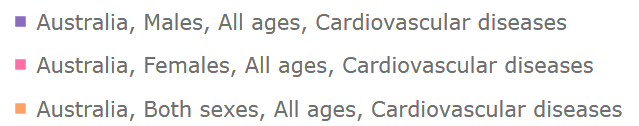


1. **IHD deaths and DALYs**


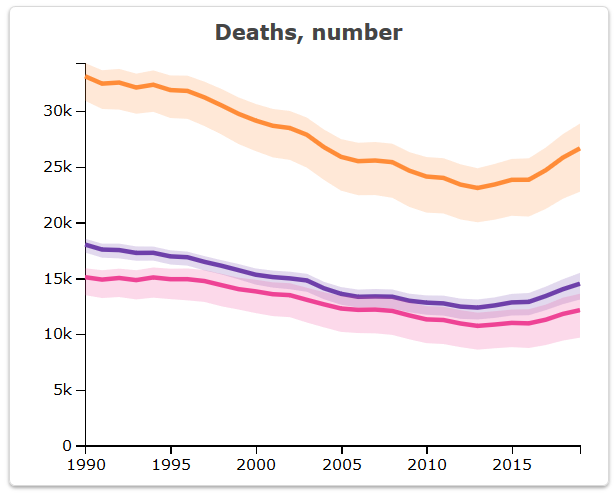

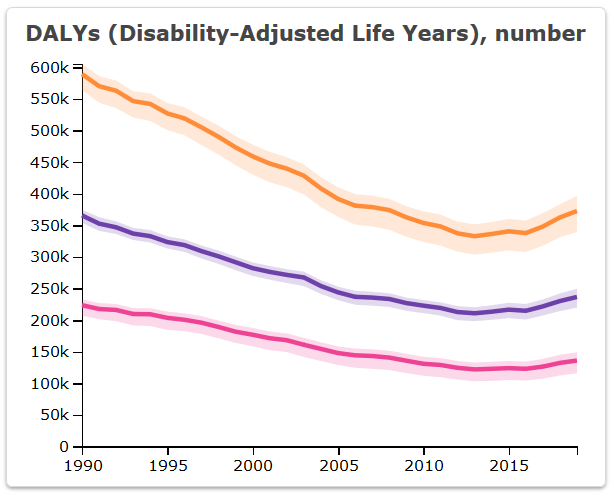


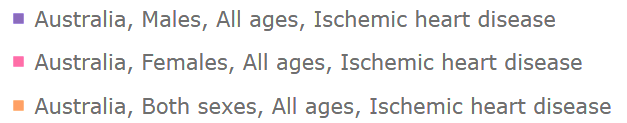


1. **Stoke deaths and DALYs**


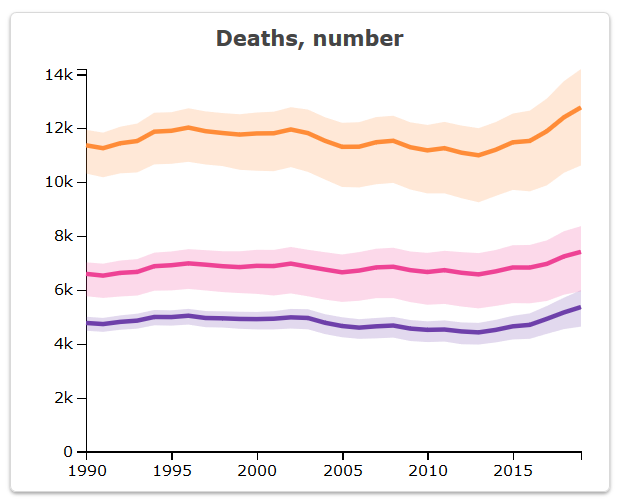

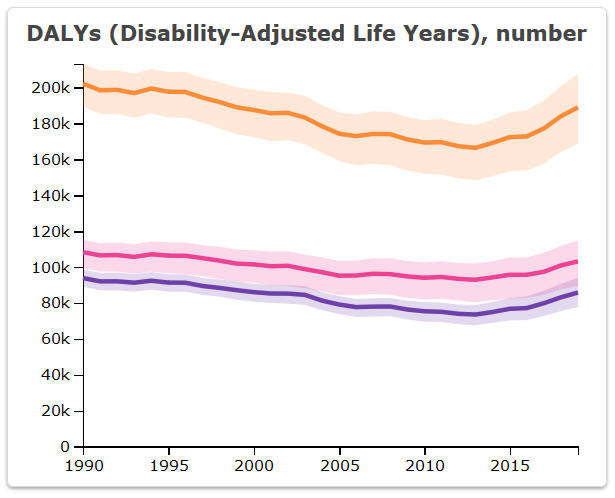


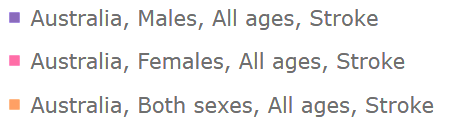


1. **Hypertensive heart disease deaths and DALYs**


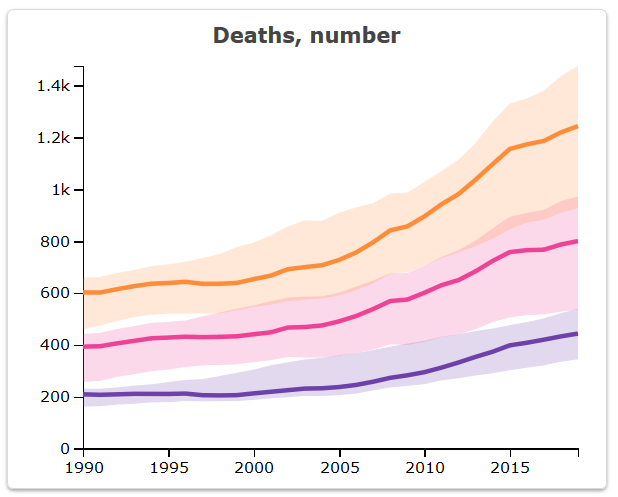

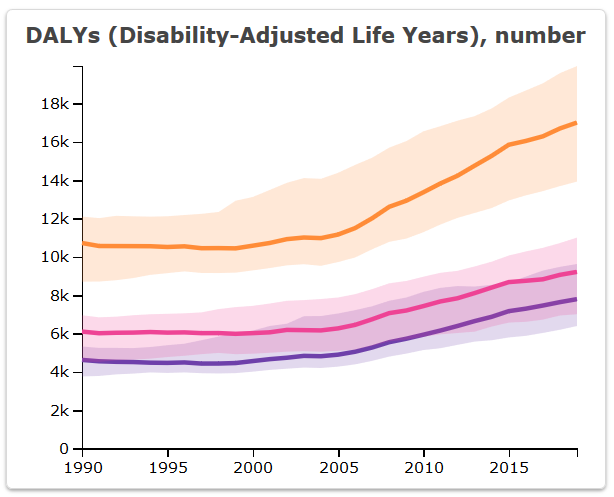


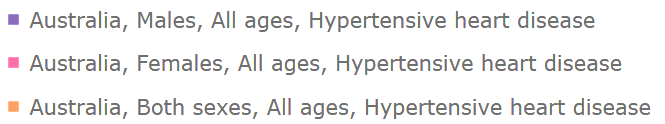


1. **AF deaths and DALYs**


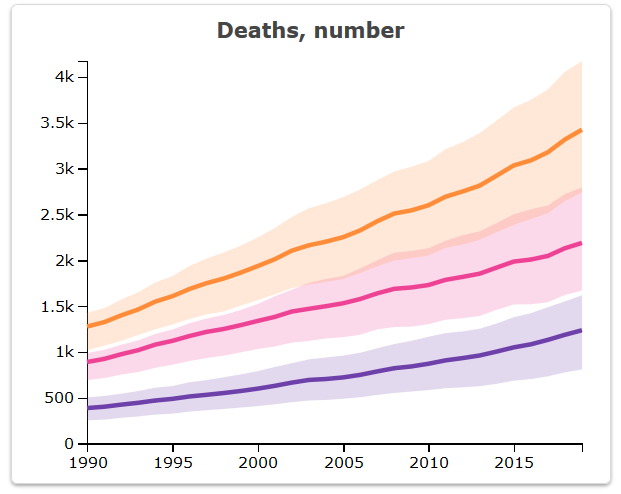

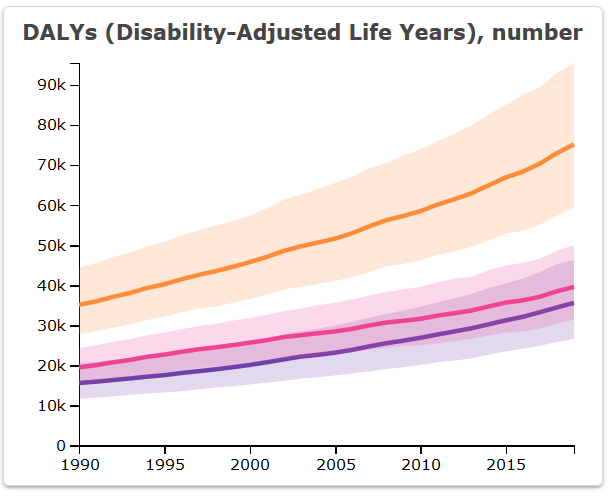


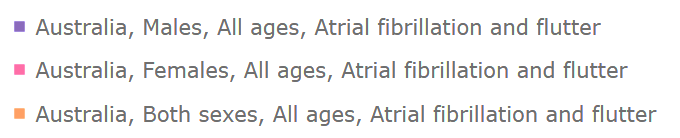


1. **PAD deaths and DALYs**


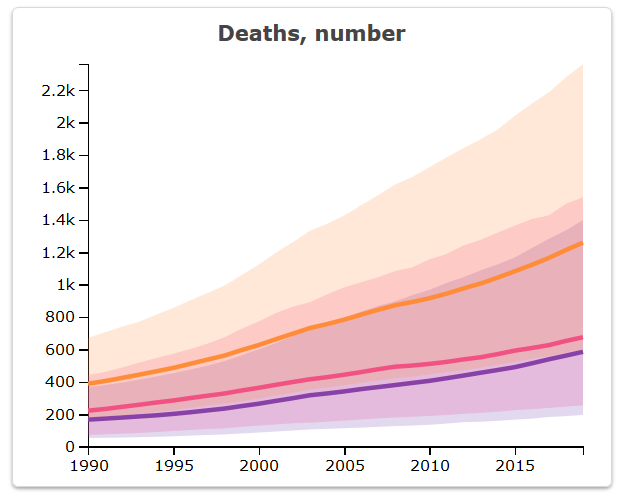

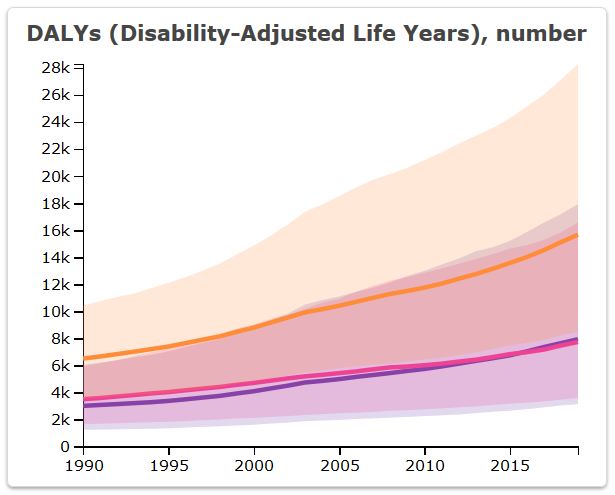


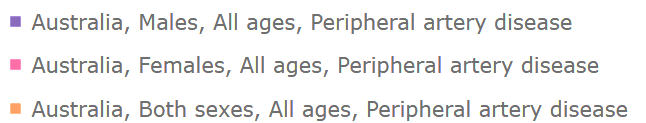


*Source from IHME. Available at:* [*https://vizhub.healthdata.org/gbd-results/*](https://vizhub.healthdata.org/gbd-results/)
