# Supplementary material for: The contribution of raised blood pressure to all-cause and cardiovascular deaths and disability-adjusted life-years (DALYs) in Australia: Analysis of global burden of disease study from 1990 to 2019
Source: PLoS One. 2024 Feb 21;19(2):e0297229. doi: 10.1371/journal.pone.0297229 (PMC10881002; doi:10.1371/journal.pone.0297229)
Supplement: S2 Fig — a No data available for the risk of high fasting plasma glucose, high LDL cholesterol, low bone mineral density, kidney dysfunction, tobacco and low physical activity for hypertensive heart disease. b No data available for the risk of high fasting plasma glucose, high LDL cholesterol, low bone mineral density, kidney dysfunction, tobacco and low physical activity for AF.c No data available for the risk of high LDL cholesterol, high BMI, low bone mineral density, alcohol use, and low physical activity for PAD. (DOCX) [file pone.0297229.s002.docx]

**Supplementary Figure 2. Number of deaths and DALYs attributable to risk factors for hypertensive heart disease, AF, and PAD in Australia between 1990 and 2019 (all ages)**

**A.** **Hypertensive heart disease deaths and DALYs^a^**

**B. AF deaths and DALYs^b^**

**C. PAD deaths and DALYs^c^**

*^a^ No data available for the risk of high fasting plasma glucose, high LDL cholesterol, low bone mineral density, kidney dysfunction, tobacco and low physical activity for hypertensive heart disease.*

*^b^ No data available for the risk of high fasting plasma glucose, high LDL cholesterol, low bone mineral density, kidney dysfunction, tobacco and low physical activity for AF.*

*^c^ No data available for the risk of high LDL cholesterol, high BMI, low bone mineral density, alcohol use, and low physical activity for PAD.*
