# Supplementary material for: The contribution of raised blood pressure to all-cause and cardiovascular deaths and disability-adjusted life-years (DALYs) in Australia: Analysis of global burden of disease study from 1990 to 2019
Source: PLoS One. 2024 Feb 21;19(2):e0297229. doi: 10.1371/journal.pone.0297229 (PMC10881002; doi:10.1371/journal.pone.0297229)
Supplement: S3 Fig — a No data on Low bone mineral density on CVD. Not include alcohol in females because it negatively contributes to CVD. b Not include alcohol because it negatively contributes to IHD. (DOCX) [file pone.0297229.s003.docx]

**Supplementary Figure 3.** **The ranking in the contribution of risk factors towards all-cause, CVD, IHD and stroke deaths in 1990, 2010, and 2019, with percentage change and 95% UI in age-standardised deaths**

**
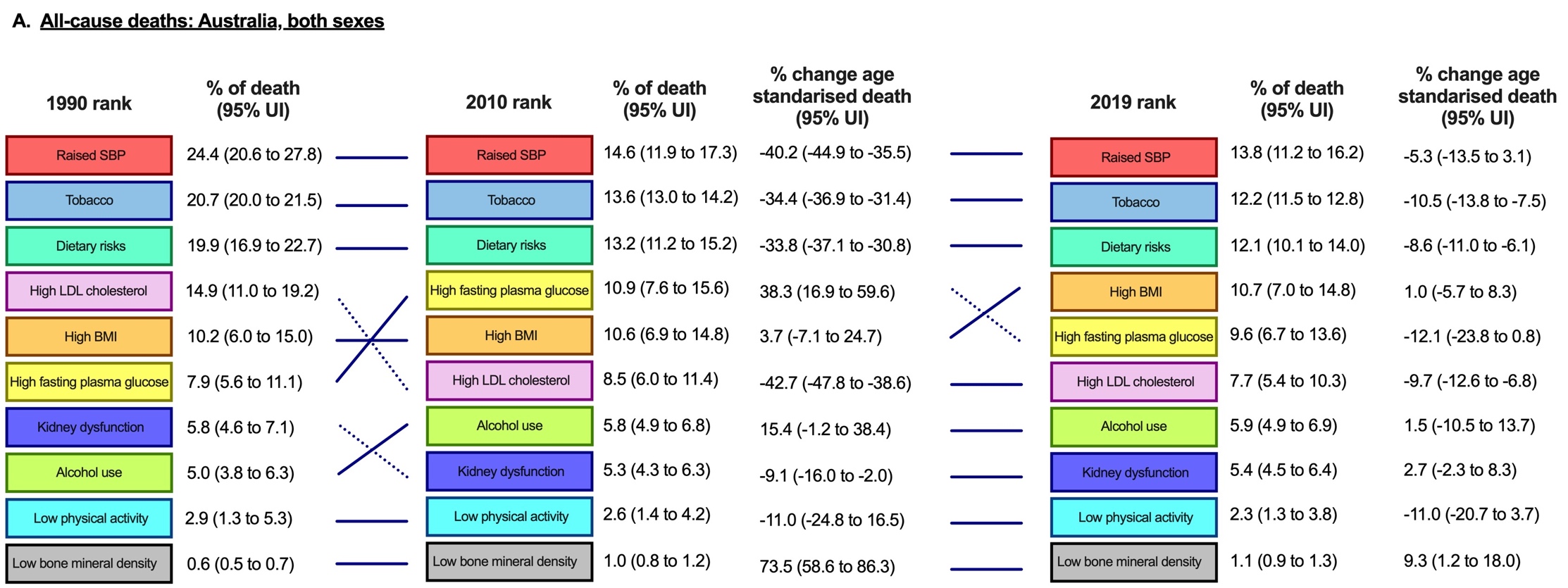
**

**
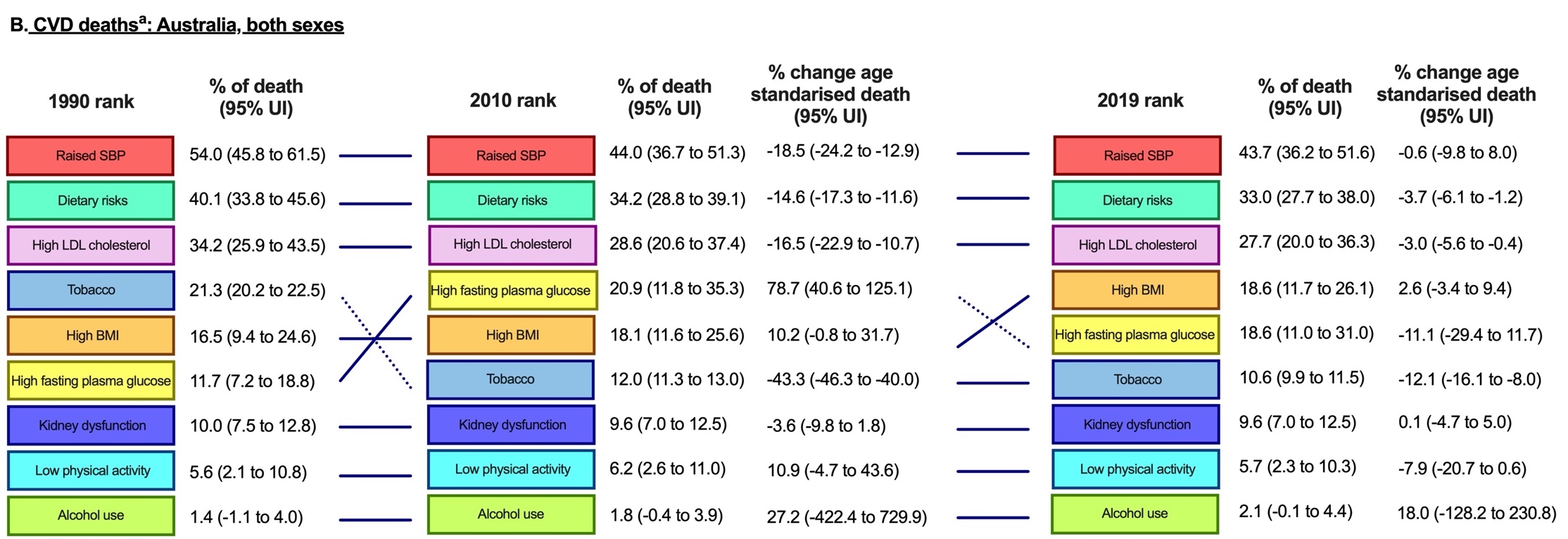
**

**
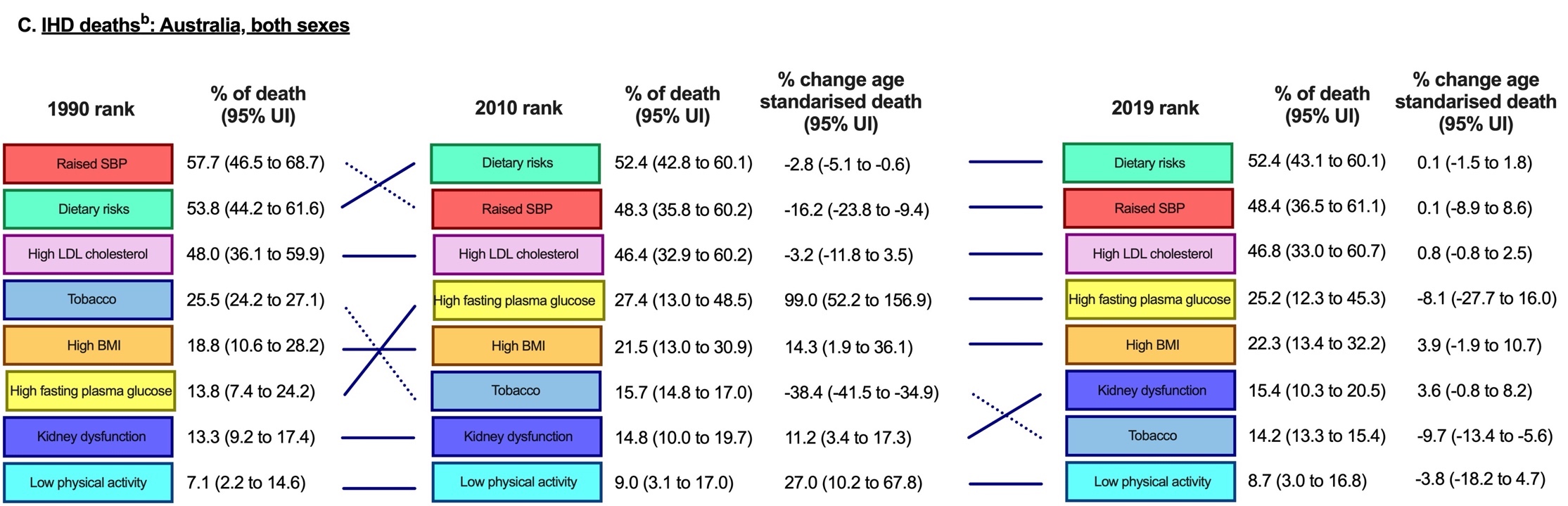
**

**
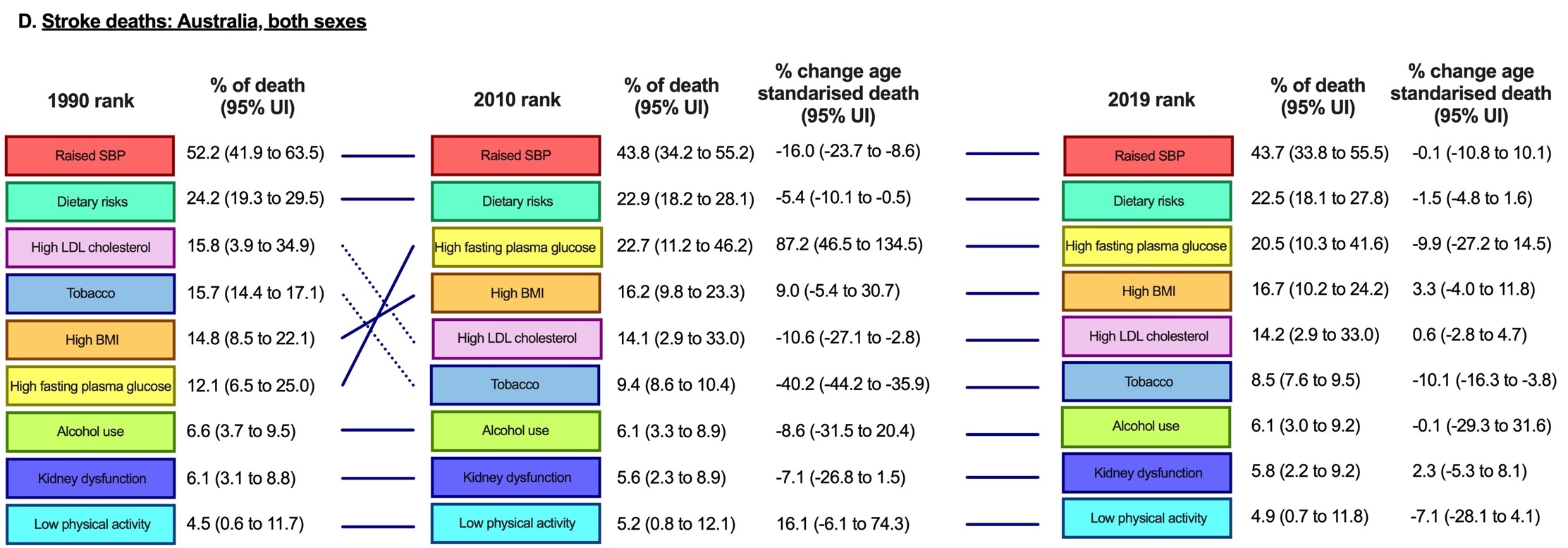
**

*^a^ No data on Low bone mineral density on CVD. Not include alcohol in females because it negatively contributes to CVD.*

***^b^*** *Not include alcohol because it negatively contributes to IHD.*
