# Supplementary material for: The contribution of raised blood pressure to all-cause and cardiovascular deaths and disability-adjusted life-years (DALYs) in Australia: Analysis of global burden of disease study from 1990 to 2019
Source: PLoS One. 2024 Feb 21;19(2):e0297229. doi: 10.1371/journal.pone.0297229 (PMC10881002; doi:10.1371/journal.pone.0297229)
Supplement: S6 Fig — *Not including aged group 10–24 years for all-cause and CVD because the value tends to be 0. No data available for males and females aged 10–24 years for IHD and stroke. (DOCX) [file pone.0297229.s006.docx]

**Supplementary Figure 6. The contribution of raised systolic blood pressure to all-cause, CVD, IHD and stroke DALYs between 2010 and 2019 by sex and age groups***


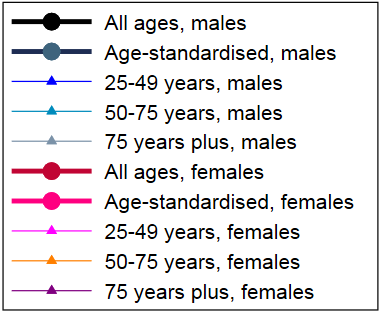

Percentage

**Not including aged group 10-24 years for all-cause and CVD because the value tends to be 0. No data available for males and females aged 10-24 years for IHD and stroke.*
