# Supplementary material for: The contribution of raised blood pressure to all-cause and cardiovascular deaths and disability-adjusted life-years (DALYs) in Australia: Analysis of global burden of disease study from 1990 to 2019
Source: PLoS One. 2024 Feb 21;19(2):e0297229. doi: 10.1371/journal.pone.0297229 (PMC10881002; doi:10.1371/journal.pone.0297229)
Supplement: S7 Fig — *No data available for males and females aged 10–24 years for AF and PAD. (DOCX) [file pone.0297229.s007.docx]

**Supplementary Figure 7. The contribution of high systolic blood pressure to AF and PAD deaths and DALYs between 2010 and 2019 by sex and age groups***

Percentage

Percentage

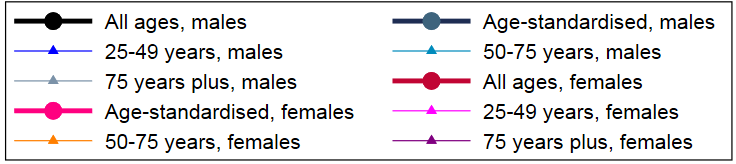


**No data available for males and females aged 10-24 years for AF and PAD.*
