# Supplementary material for: The contribution of raised blood pressure to all-cause and cardiovascular deaths and disability-adjusted life-years (DALYs) in Australia: Analysis of global burden of disease study from 1990 to 2019
Source: PLoS One. 2024 Feb 21;19(2):e0297229. doi: 10.1371/journal.pone.0297229 (PMC10881002; doi:10.1371/journal.pone.0297229)
Supplement: S1A Table — *Data source: https://www.aihw.gov.au/reports/burden-of-disease/interactive-data-risk-factor-burden/contents/overview. (DOCX) [file pone.0297229.s008.docx]

**Supplementary Table 1A. Proportion of disease DALY due to top risk factors, Australia Burden of Disease Study 2015***

| **Risk factors** | **All cause DALY** | **CVD DALYs** |
| --- | --- | --- |
| Tobacco | 9.3 | 11.5 |
| High BMI | 8.4 | 19.3 |
| Dietary risks | 7.3 | 40.2 |
| High blood pressure | 5.8 | 38.0 |
| High blood plasma glucose | 4.7 | 4.9 |
| Alcohol use | 4.5 | 3.6 |
| High cholesterol | 3.0 | 21.8 |
| Illicit drug use | 2.7 | - |
| Low physical activity | 2.5 | 8.0 |
| Child abuse & neglect | 2.2 | - |
| Kidney function | 2.1 | 3.9 |

**Data source:* [*https://www.aihw.gov.au/reports/burden-of-disease/interactive-data-risk-factor-burden/contents/overview*](https://www.aihw.gov.au/reports/burden-of-disease/interactive-data-risk-factor-burden/contents/overview)
