# Supplementary material for: The contribution of raised blood pressure to all-cause and cardiovascular deaths and disability-adjusted life-years (DALYs) in Australia: Analysis of global burden of disease study from 1990 to 2019
Source: PLoS One. 2024 Feb 21;19(2):e0297229. doi: 10.1371/journal.pone.0297229 (PMC10881002; doi:10.1371/journal.pone.0297229)
Supplement: S1B Table — *Data source: https://www.aihw.gov.au/reports/burden-of-disease/interactive-data-risk-factor-burden/contents/diseases-and-associated-risk-factors. (DOCX) [file pone.0297229.s009.docx]

**Supplementary Table 1B. Proportion of each CVD DALY due to risk factors by sex, Australia Burden of Disease Study 2015***

| **Risk factors** | **Coronary heart disease** | | **Stroke** | | **Hypertensive heart disease** | | **AF** | | **PAD** | |
| --- | --- | --- | --- | --- | --- | --- | --- | --- | --- | --- |
|  | **Men** | **Women** | **Men** | **Women** | **Men** | **Women** | **Men** | **Women** | **Men** | **Women** |
| Dietary risks | 64.0 | 58.3 | 37.6 | 31.0 | 11.4 | 7.7 | 8.9 | 6.5 | 6.4 | 4.8 |
| High blood pressure | 43.7 | 42.8 | 41.7 | 40.1 | 64.9 | 65.0 | 33.1 | 31.2 | 23.4 | 22.3 |
| High cholesterol | 38.9 | 33.5 | 15.6 | 14.3 | - | - | - | - | - | - |
| High BMI | 27.8 | 18.3 | 24.9 | 17.3 | 51.4 | 38.8 | 28.4 | 24.8 | - | - |
| Tobacco | 15.8 | 10.4 | 12.1 | 9.7 | 16.0 | 6.3 | 11.2 | 5.4 | 10.3 | 7.2 |
| Low physical activity | 12.0 | 11.3 | 10.5 | 10.2 | - | - | - | - | - | - |
| Air population | 7.4 | 7.4 | 4.1 | 4.1 | - | - | - | - | - | - |
| High blood plasma glucose | 6.6 | 6.9 | 6.2 | 6.0 | - | - | - | - | 20.2 | 14.3 |
| Kidney function | 4.9 | 7.8 | 3.4 | 4.1 | - | - | - | - | 10.1 | 8.9 |
| Alcohol use | 2.5 | 5.4 | 7.1 | 3.7 | 12.8 | 5.0 | 9.7 | 8.8 | - | - |

**Data source:* [*https://www.aihw.gov.au/reports/burden-of-disease/interactive-data-risk-factor-burden/contents/diseases-and-associated-risk-factors*](https://www.aihw.gov.au/reports/burden-of-disease/interactive-data-risk-factor-burden/contents/diseases-and-associated-risk-factors)
